# Longitudinal Suicide Risk in Children and Adolescents With Attention Deficit and Hyperactivity Disorder: A Systematic Review and Meta‐Analysis

**DOI:** 10.1002/brb3.70618

**Published:** 2025-06-18

**Authors:** Peter Garas, Zsofia K. Takacs, Judit Balázs

**Affiliations:** ^1^ Mental Health Sciences School Semmelweis University Budapest Hungary; ^2^ School of Health in Social Science the University of Edinburgh Edinburgh UK; ^3^ Institute of Psychology Eötvös Loránd University Budapest Hungary; ^4^ Oslo New University College Oslo Norway

**Keywords:** ADHD, adolescent, children, longitudinal study, suicidal behavior

## Abstract

**Background/Aims:**

Attention‐deficit/hyperactivity disorder (ADHD) is one of the most common mental disorders among young people and significantly affects their quality of life. Previous research suggests an increased risk of suicidal behavior among individuals with ADHD; however, this has not yet been investigated in a meta‐analysis of long‐term studies. The primary aim of this study was to conduct an updated systematic review of longitudinal studies on ADHD and suicidality supplemented by meta‐analytic calculations.

**Methods:**

A systematic search was conducted across the following databases: OVID Medline, OVID PsychInfo, PubMed, Scopus, and Web of Science. Only longitudinal studies were included, in which most participants were under 18 years of age at baseline, had a clinical ADHD diagnosis, and provided sufficient data about suicidal behavior.

**Results:**

In total, nine studies were included in both the meta‐analysis and narrative review. The average odds ratios were significant and small to moderate in size for the following aspects: overall suicidality (OR = 3.336, 95% CI: 2.201; 5.057, *p* < 0.001), suicidal ideation (OR = 3.956, 95% CI: 1.996; 7.841, *p* < 0.001), suicide attempt (OR = 3.344, 95% CI: 1.682; 6.650, *p* = 0.001), and death (OR = 3.891, 95% CI: 2.103; 7.198, *p* < 0.001). The number of participants with ADHD ranged from 104 to 86,670, with a mean age between 5.2 and 14.94 years old, and the majority were male. Suicide behavior was more common in the ADHD combined subtype and the hyperactive/impulsive subtype. There are conflicting results regarding sex differences and the role of comorbidities.

**Conclusions:**

The current systematic review and meta‐analysis confirms previous findings that individuals with ADHD are at an elevated risk for suicidal behavior. However, this relationship is heterogeneous and complex, with significant differences across ADHD subtypes, age groups, sexes, comorbidities, and social issues, all of which play important roles in the development of suicidal behavior.

## Graphical abstract

The aim was an updated systematic review of longitudinal studies on ADHD and suicidality supplemented by meta‐analytic calculationsIn total, nine studies were included in both the meta‐analysis and narrative review. The average odds ratios were significant and small to moderate in size for overall and specific suicidal behavior. The current systematic review and meta‐analysis confirms previous findings that individuals with ADHD are at an elevated risk for suicidal behavior.

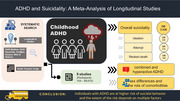

## 1 Introduction

Sith a prevalence rate of 5.9%–14%, attention‐deficit/hyperactivity disorder (ADHD) is one of the most common mental disorders among children and adolescents (Faraone et al. 2015). The estimated prevalence is more than twice as high in children aged between 6 and 11 years old (7%) than in adolescents aged between 12 and 18 years old (3%) (Polanczyk et al. 2015). ADHD is more common in males, presenting with a sex ratio of 2:1 (Faraone et al. 2015; Ayano et al. 2023). The core symptoms of ADHD are hyperactivity/impulsivity and inattention, and the diagnosis requires the presence of symptoms before the age of 12 (Association 2013). The most common subtype is the ADHD inattentive (ADHD‐I) subtype in both males and females, followed by the hyperactive (ADHD‐H) and combined (ADHD‐C) subtypes (Ayano et al. 2023). Depending on the severity of symptoms, ADHD has a significant negative effect on quality of life, including social problems (Bagwell et al. 2001), educational problems (Wilson and Marcotte 1996), and criminality (Gudjonsson et al. 2014). The most common comorbidities that develop in adolescence are major depression, conduct disorders, and anxiety disorders (Taurines et al. 2010), and an increased risk of substance abuse (Carpentier et al. 2012). Treatment is more challenging in the presence of comorbidities (Faraone et al. 2015).

Suicidality is the second most common cause of death among those aged 15–29 years old (World Health Organization 2016). In a recent meta‐analysis, Liu et al. (2022) reported that ADHD is the second strongest risk factor for suicidal ideation, with a medium effect size, following major depression; the authors specifically highlighted the risk for suicidal thoughts in the preadolescent ADHD population. Recently, several reviews and meta‐analyses focused on ADHD and suicidality, revealing increasing evidence for a connection between ADHD and suicidal behavior (i.e., attempts, ideations, plans, and completed suicide) in all sexes and age groups (James et al. 2004; Nigg 2013; Balazs and Kereszteny 2017; Impey and Heun 2012; Septier et al. 2019; Giupponi et al. 2018; Austgulen et al. 2023). However, the exact relationship (e.g., considering the role of comorbidities) between ADHD and suicidal behavior is still unclear (James et al. 2004; Balazs and Kereszteny 2017).

Although some of these previous reviews focused on the child and adolescent population, none specifically examined the relationship from a longitudinal perspective. Additionally, no previous reviews included only samples with a clinical diagnosis of ADHD.

To our knowledge, the only meta‐analysis that focused on the relationship between suicidal behavior and ADHD was conducted by Septier et al. 16 In this study, meta‐analytic calculations were conducted based on cross‐sectional study data. As the authors also noted, these results do not confirm the cause‐and‐effect relationship or even temporal precedence. Additionally, it should be noted that the majority of the 57 databases included in the analysis examined adult or mixed populations, and results regarding those under 18 were limited.

In summary, the current evidence base is limited, and no meta‐analytic results are available on the long‐term association between clinical ADHD in childhood and adolescence on the one hand and later suicide behavior on the other.

Garas and Balazs (2020) performed a scientific search for a systematic review on the longitudinal relationship between ADHD and suicidality 5 years ago; the majority of the selected papers confirmed a greater suicide risk in youth with ADHD; however, no meta‐analytic calculations have been conducted to estimate the size of the effect. It is important to note that the selected papers were heterogeneous in terms of methodology, such as ADHD diagnostic tools, measures of suicidal behavior, monitoring treatment, and assessing comorbidities, especially major depression. To the best of our knowledge, no meta‐analysis has investigated the longitudinal relationship between ADHD and suicidal behavior in children and adolescents. Thus, the goal of the present study was to provide an updated systematic review of longitudinal studies on ADHD and suicidality (Garas and Balazs 2020) and to supplement this synthesis with meta‐analytic calculations.

## 2 Methods

### 2.1 Study Selection

The systematic search for eligible papers was conducted on July 18, 2024, in the following electronic scientific databases: OVID Medline, OVID PsychInfo, PubMed, Scopus, and Web of Science. We used the same search terms as those in the systematic review of Garas and Balazs (Garas and Balazs 2020): (ADHD OR attention deficit hyperactivity disorder) AND (suicide OR suicidal OR suicidality) AND (follow‐up OR longitudinal study OR prospective study), and we filtered for publications in the past 5 years. Thus, in addition to the articles included in the previous systematic review, we screened publications published in the past 5 years to update the systematic search. We used the software Zotero Ver 6.0.37 to remove duplicates and for screening. We selected the eligible studies using the following criteria: (1) They were written in English. (2) The majority of participants were under 18 years at baseline; 3. They had a longitudinal design (including birth cohort studies); 4. An ADHD sample diagnosed in childhood was followed up for suicidal behavior; 5. The study included a control group without ADHD being followed up for suicidal behavior; 6. The ADHD group was diagnosed with ADHD based on the DSM‐5 criteria; and 7. The number of participants in total and those with suicidal behavior in both the ADHD group and control group during follow‐up were reported. We excluded reviews and meta‐analyses, book chapters, guidelines, letters, dissertations, and studies that aimed to investigate drug treatment efficacy in ADHD patients. If there was more than one study reporting on the same sample, we selected the one with the largest sample.

**FIGURE 1:**
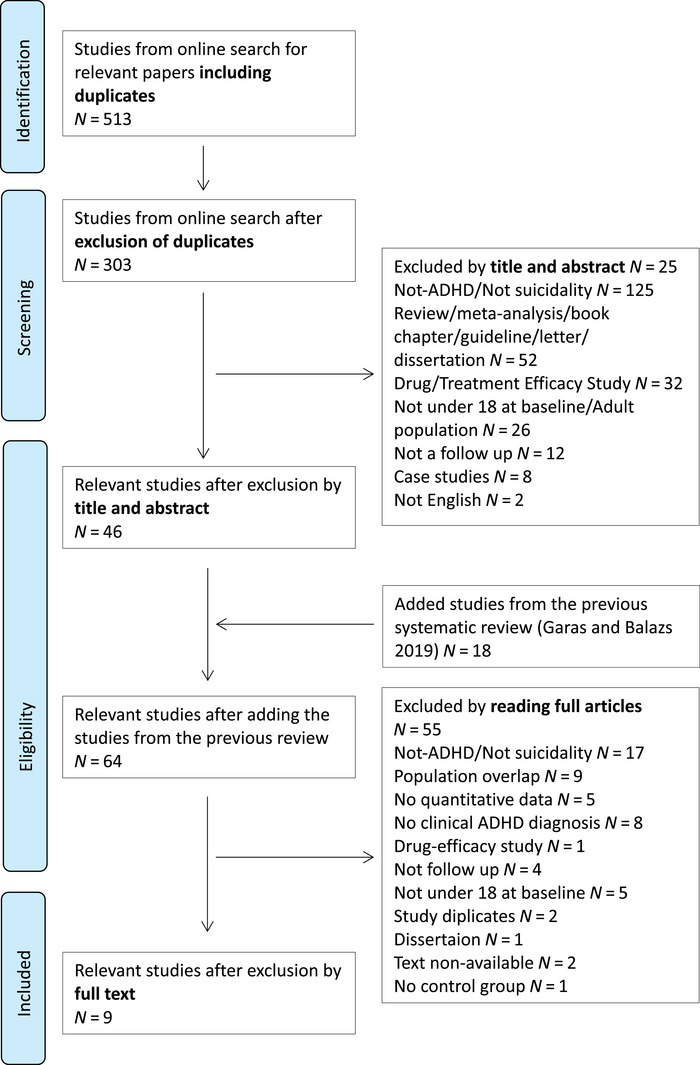
PRISMA flowchart of the selection method.

After excluding duplicates, the first author screened the studies based on the title and abstract. After the papers included in the previous systematic review (Garas and Balazs 2020) were added. The rationale for including studies from the previous review was as follows: First, in the previous review, we applied exactly the same standard procedure in the first two steps (i.e., using the same search terms and screening studies based on title and abstract). Second, there were only two differences in our inclusion criteria: we only included studies with data regarding the longitudinal association between the two concepts and only studies with a DSM‐5‐based diagnosis of ADHD in childhood. The studies were further screened based on the full texts of the articles by both the first author and a research assistant, independently. The studies were further screened based on the full texts of the articles by the first author and by a research assistant independently. If the suggestions were not in line with each other, the third author made the final suggestion based on the reading of the full‐text article. The selection method is summarized in the PRISMA flowchart (Figure 1).

**FIGURE 2:**
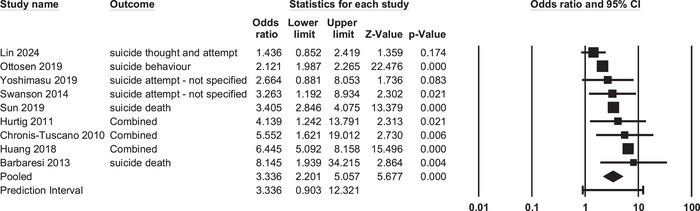
Forest plot of the nine studies with odds ratios for suicidal behavior in ADHD and comparison samples.

### 2.2 Coding and Data Extraction

The following information was extracted from the eligible studies: bibliographic data (e.g., first author, year of publication, title, publication type); characteristics of the ADHD sample (e.g., country and continent, mean age, sex ratio, socioeconomic status [SES], what the ADHD diagnosis was based on, whether previous suicidal behavior and comorbid conditions were assessed at baseline, whether comorbid conditions were assessed at follow‐up, what comorbidities were reported, whether ADHD diagnosis was confirmed at follow‐up, data about medication, ratio of ADHD subtypes); characteristics of the comparison sample (e.g., whether they were matched to the ADHD group in terms of age and sex); design (e.g., follow‐up time, drop‐out rate); and measure(s) of suicidal behavior (e.g., whether suicidal ideation, attempt or death was measured, based on self‐reports or medical reports, in what period it was assessed, and whether a validated or nonvalidated scale or a single item was used).

### 2.3 Quality Assessment

The selected studies were assessed via the Joanna Briggs Institute (JBI) Critical Appraisal Checklist for Cohort Studies (JBI n.d.), which contains 11 criteria. Each criterion has four options: yes, no, unclear, and not applicable. One point was given for each criterion that was met, resulting in a maximum score of 11 points, with higher scores reflecting higher quality. The evaluation was carried out by the first author.

### 2.4 Quantitative Data Analysis

The effect size in the present study was the odds ratio of suicide behavior following ADHD diagnosis in childhood, here based on the frequencies of the participants in four groups: 1. the subgroup that presented suicidal behavior from the ADHD sample; 2. the total ADHD sample; 3. the subgroup that presented suicidal behavior from the comparison group; and 4. the total comparison group. We used Comprehensive Meta‐Analysis software (v4) for the analyses. An odds ratio above 1 indicates an elevated risk of suicidal behavior in the ADHD group compared with the comparison group. We interpreted odds ratios up to 3.47 as small and those larger than 6.71 as large (Chen et al. 2010).

Three of the nine eligible provided data for more than one suicide behavior variable, which was a mix of suicide attempt and suicide ideation in two cases and two different measures of suicide attempt (first or repeated) in one study. The average odds ratios were calculated by the software before the studies were included in the meta‐analytic analyses.

Because of the heterogeneity of the primary studies, a random effects model was used to calculate an average effect size. The standardized residuals were inspected to identify potential outlying studies (exceeding ± 3.29). Heterogeneity was assessed by the *Q* statistic and *I* ^2^. Publication bias was investigated by inspecting the funnel plot and Egger's test. In addition, the Duval and Tweedie trim and fill procedure was applied to calculate an adjusted average effect size. The results were also pooled separately for the different specific suicidal behaviors reported in the primary studies: (1) suicidal ideation, (2) suicide attempt, and (3) suicidal death. This was decided on because, although these belong to the broad concept of suicidal behavior, they measure very different levels of that behavior.

## 3 Results

After the literature search and exclusion of duplicates, 303 papers underwent the screening phase. In the eligibility phase, we added 18 papers included in the previous systematic search (Garas and Balazs 2020). After the selection process, nine papers were included in the meta‐analysis and systematic review (Chronis‐Tuscano et al. 2010; Hurtig et al. 2012; Huang et al. 2018; Swanson et al. 2012; Lin et al. 2024; Ottosen et al. 2019; Yoshimasu et al. 2019; Barbaresi et al. 2013; Sun et al. 2019). After quality assessment, all selected studies were rated eight points or more from the maximum of 11 points. However, there were three common issues that negatively affected the quality of the articles: it was unclear whether suicidal behavior occurred in the past; some prospective studies lacked information on drop‐out rates and strategies for handling missing data due to drop‐out. The results of the quality assessment of the nine included studies are shown in Table 1.

**TABLE 1:** Quality assessment of selected articles.

Chronis‐Tuscano et al. (2010)	Yes	Yes	Yes	Yes	Yes	Unclear	Yes	Yes	Yes	Yes	Yes	0	10
Huang et al. (2018)	Yes	Yes	Yes	Yes	Yes	Yes	Yes	Yes	NA	NA	Yes	2	9
Hurtig et al. (2011)	Yes	Yes	Yes	Yes	Yes	Unclear	Yes	Yes	Yes	Yes	Yes	0	10
Swanson et al. (2014)	Yes	Yes	Yes	Yes	Yes	Unclear	Yes	Yes	No	No	Yes	0	8
Lin et al. (2024)	Yes	Yes	Yes	Yes	Yes	Unclear	Yes	Yes	NA	NA	Yes	2	8
Ottosen et al. (2019)	Yes	Yes	Yes	Yes	Yes	Unclear	Yes	Yes	NA	NA	Yes	2	9
Sun et al. (2019)	Yes	Yes	Yes	Yes	Yes	NA	Yes	Yes	NA	NA	Yes	3	8
Yoshimasu et al. (2019)	Yes	Yes	Yes	Yes	Yes	Unclear	Yes	Yes	No	No	Yes	0	8
Barbaresi et al. (2013)	Yes	Yes	Yes	Yes	Yes	Unclear	Yes	Yes	NA	NA	Yes	2	8
	Were the two groups similar and recruited from the same population?	Were the exposures measured similarly to assign people to both exposed and unexposed groups?	Was the exposure measured in a valid and reliable way?	Were confounding factors identified?	Were strategies to deal with confounding factors stated?	Were the groups/participants free of the outcome at the start of the study (or at the moment of exposure)?	Were the outcomes measured in a valid and reliable way?	Was the follow up time reported and sufficient to be long enough for outcomes to occur?	Was follow up complete, and if not, were the reasons to loss to follow up described and explored?	Were strategies to address incomplete follow‐up utilized?	Was appropriate statistical analysis used?	No. of NA answers	Summary

### 3.1 Descriptive Results

Descriptive information regarding the included studies is shown in Table 1. The nine studies were published between 2010 and 2024. The number of participants with ADHD was between 104 and 86,670, whereas the number of participants in the comparison group was between 88 and 2,588,945 in the primary studies. The geological distribution of the selected studies was as follows: three were from Europe (Finland [Hurtig et al. 2012], Denmark [Ottosen et al. 2019], and Sweden [Sun et al. 2019]), three were from North America (all from the United States [Chronis‐Tuscano et al. 2010; Swanson et al. 2012; Yoshimasu et al. 2019]), one was from Australia (Lin et al. 2024), and one was from Asia (Taiwan [Huang et al. 2018]). The key characteristics of the selected articles are shown in Table 2.

**TABLE 2:** The key characteristic of the selected articles.

Reported ADHD subtypes at Baseline	Yes (ADHD‐C, ‐I, ‐HI)		No	No	No	Yes (ADHD‐C, ADHD‐I)	No	No	No	No	No
**% of males**	81,3		78,8	NA		0	50,6	50,92	62,70%	62,70%	NA
**Mean age (SD)**	5,2 (0,8)		14,94 (3,49)	8 (NA)		9,43 (1,65)	10 (NA)	NA	NA	NA	NA
**What period does suicide data refer to?**	since Baseline	1‐12 months	since Baseline	12+ months	12+ months	lifetime	1‐12 months	since Baseline	since Baseline	lifetime	since Baseline
**Suicide measurement**	Self‐report	Self‐report	Based on medical report	Interview	Interview	Self‐report	Self‐report	Based on medical report	Based on medical report	Interview	Based on medical report
**Suicide sample size from the control group at follow‐up**	2	7	333	40	2	5	330	23,196	1168	5	5
**Suicide sample size from ADHD group at follow‐up**	15	23	546	53	6	23	17	958	133	9	3
**Sample size (at follow‐up) healthy control at baseline**	123	123	61,722	169	169	88	3563	1,633,421	2,588,945	335	4946
**Sample size (at follow‐up) with ADHD at baseline**	125	125	20,574	104	104	140	133	32,308	86 670	232	367
**Type of suicide data**	Suicide Ideation	Suicide Attempt	Suicide Attempt	Suicide Ideation	Suicide Attempt	Suicide Attempt	Suicide Attempt, Suicide Ideation	Suicide Attempt, Suicide Ideation	Suicide death	Suicide Attempt	Suicide Death
**Follow‐up time (in months)**	168		24–132	84–96		120	48	264	133,2 (SD 32,7)	NA	264–324
**Source of the population data**	USA		Taiwan	Finnland		Canada	Australia	Denmark	Sweden	USA	USA
**Sample type**	clinical sample		birth cohort	normal sample		normal sample	birth cohort	birth cohort	birth cohort	birth cohort	birth cohort
**Author**	Chronis‐Tuscano et al. (2010) *		Huang et al. (2018) *	Hurtig et al. (2011) *		Swanson et al. (2014) *	Lin et al. (2024)	Ottosen et al. (2019)	Sun et al. (2019)	Yoshimasu et al. (2019) *	Barbaresi et al. (2013) *

Abbreviations: ADHD‐C, attention‐deficit/hyperactivity disorder combines subtype; ADHD‐I: ADHD inattentive subtype; ADHD‐HI: ADHD hyperactive subtype.

*Articles selected in the previous review by Garas and Balazs (2020).

### 3.2 Samples

Six of the nine selected studies used data from retrospective birth cohorts (Huang et al. 2018; Lin et al. 2024; Ottosen et al. 2019; Yoshimasu et al. 2019; Barbaresi et al. 2013; Sun et al. 2019). The mean age of the ADHD group at baseline ranged between 5.2 and 14.94 years old. The sex proportion of the ADHD population was mainly males (> 65%) in seven of the nine studies (Chronis‐Tuscano et al. 2010; Huang et al. 2018; Lin et al. 2024; Ottosen et al. 2019; Yoshimasu et al. 2019; Barbaresi et al. 2013; Sun et al. 2019), whereas only one study focused on a female‐only sample (Swanson et al. 2012), and the sex ratio was not reported in one study. The sex proportion and the mean age of the control group were matched to those of the ADHD group in only three studies.

The SES of the sample (i.e., income, educational level, or both) was reported in seven studies (Chronis‐Tuscano et al. 2010; Huang et al. 2018; Lin et al. 2024; Ottosen et al. 2019; Yoshimasu et al. 2019; Barbaresi et al. 2013; Sun et al. 2019), two of which reported data about both family income and paternal education level (Lin et al. 2024; Ottosen et al. 2019), and all seven studies investigated a mixed SES population. Only two studies investigated ADHD subtypes, both of which reported that the majority of the sample (> 65%) had a diagnosis of the combined subtype (Chronis‐Tuscano et al. 2010; Swanson et al. 2012).

### 3.3 Follow‐Up Period

The follow‐up time in the nine studies varied between 4 and 27 years. Three studies reported medication data at the follow‐up in the ADHD group (Huang et al. 2018; Swanson et al. 2012; Lin et al. 2024) and found that 42%–60% of the ADHD participants received medication. Only one focused on the effect of the medication status, where a decreasing effect of long‐term methylphenidate treatment for repeated suicide attempts in men was reported (Huang et al. 2018). Importantly, among the three prospective cohort studies (Chronis‐Tuscano et al. 2010; Hurtig et al. 2012; Swanson et al. 2012), two confirmed the ADHD diagnosis at follow‐up (Hurtig et al. 2012; Swanson et al. 2012).

### 3.4 Suicidal Measurement

Two articles examined suicide ideation (Chronis‐Tuscano et al. 2010; Hurtig et al. 2012), six examined suicide attempts (Chronis‐Tuscano et al. 2010; Hurtig et al. 2012; Huang et al. 2018; Swanson et al. 2012; Lin et al. 2024; Yoshimasu et al. 2019), and two examined suicidal death (Barbaresi et al. 2013; Sun et al. 2019). To evaluate suicidal thoughts and attempts, four studies used medical records (Huang et al. 2018; Ottosen et al. 2019; Barbaresi et al. 2013; Sun et al. 2019), three studies used self‐reports (Chronis‐Tuscano et al. 2010; Swanson et al. 2012; Lin et al. 2024), and two studies used interviews (Hurtig et al. 2012; Yoshimasu et al. 2019). In three studies, suicidality status was measured with only two or a single self‐report questionnaire or interview items (Chronis‐Tuscano et al. 2010; Hurtig et al. 2012; Lin et al. 2024).

## 4 Quantitative Synthesis

### 4.1 Overall Suicidal Behavior

There were no outlying effect sizes among the nine studies. The total number of participants with ADHD in the nine studies was 140,654, and the total number of participants in the comparison groups was 4,293,312. As shown in Figure 2, the effect sizes ranged widely between 1.436 (very small) and 8.145 (large). The average odds ratio was significant and small to moderate in size (OR = 3.336, 95% CI: 2.201; 5.057, *p* < 0.001). This effect was highly heterogeneous (*Q* (8) = 105.17, *p* < 0.001, *I* ^2^ = 92.39).

The results regarding potential publication bias were mixed. The funnel plot showed a somewhat asymmetrical distribution, but Egger's test was not significant (*p* = 0.22). When we included the one imputed study that Duval and Tweedie's trim and fill procedure estimated, the adjusted average effect size remained similar in size and still significant (OR_adj_ = 3.147, 95% CI: 2.103; 4.709). Thus, publication bias does not seem evident here.

As a sensitivity analysis, we calculated the mean odds ratio excluding suicidal death. This produced a similar, significant mean effect (OR = 3.127, 95% CI: 1.748; 5.594, *p* < 0.001) in the seven studies. Heterogeneity was still high (*Q* (6) = 85.69, *p* < 0.001, *I* ^2^ = 93.00).

### 4.2 Suicide Ideation

Two studies reported on the prevalence of suicide ideation specifically (Chronis‐Tuscano et al. 2010; Hurtig et al. 2012). These variables had a moderate‐sized, significant average odds ratio (OR = 3.956, 95% CI: 1.996; 7.841, *p* < 0.001). This was a homogeneous effect (*Q* (1) = 1.24, *p* = 0.27, *I* ^2^ = 19.25).

### 4.3 Suicide Attempt

Six studies reported on the prevalence of suicide attempts specifically (Chronis‐Tuscano et al. 2010; Hurtig et al. 2012; Huang et al. 2018; Swanson et al. 2012; Lin et al. 2024; Yoshimasu et al. 2019). These factors had a small‐to‐moderate, significant average effect (OR = 3.344, 95% CI: 1.682; 6.650, *p* = 0.001). This effect was heterogeneous (*Q* (5) = 28.54, *p* < 0.001, *I* ^2^ = 82.48).

### 4.4 Suicide Death

Two studies reported on the prevalence of suicide death specifically (Barbaresi et al. 2013; Sun et al. 2019). These results revealed a significant moderate‐sized average effect (OR = 3.891, 95% CI: 2.103; 7.198, *p* < 0.001). This effect was homogeneous (*Q* (1) = 1.40, *p* = 0.24, *I* ^2^ = 28.38).

We also conducted a subgroup analysis to compare ORs in studies with short (< 10 years) and longer (≥ 10 years) follow‐up, as per reviewer feedback. We had to exclude two studies from this analysis: one because this information was not reported (Yoshimasu et al. 2019) and one that could not be categorized because follow‐up time ranged from 2 to 11 years (Huang et al. 2018). This resulted in two studies with a short follow‐up time producing a non‐significant small average effect (OR = 2.110, 95% CI: 0.777; 5.724, *p* = 0.14) and five studies with a long follow‐up time that showed a significant average effect (OR = 3.095, 95% CI: 1.126; 8.504, *p* = 0.028).

After the comparison of the ORs in terms of the different method of evaluation of the suicide thoughts and attempts, the average effect size was similar in size with overlapping confidence intervals and significant in all three types of studies (medical record: OR = 3.898, 95% CI: 2.175; 6.985, *p* < 0.001; self‐report: OR = 2.514, 95% CI: 1.121; 5.636, *p* = 0.025; clinical interview: OR = 3.260, 95% CI: 1.444; 7.360, *p* = 0.004).

## 5 Narrative Review of Other Factors Associated With ADHD and Suicidality

There was a high level of heterogeneity in the meta‐analytic estimates, and further investigations into other factors, such as sex differences, ADHD subtypes, and comorbidities, are warranted. Considering the relatively small number of included studies, we could not conduct moderator analyses. Thus, to investigate the potential moderating role of the characteristics of the samples and research methodology in the primary studies, a qualitative synthesis was used.

### 5.1 Sex Differences

Four studies published data regarding sex differences in suicidality in the ADHD population. Among these studies, the proportion of male participants was high in the ADHD groups (between 71.78% and 85.6%). One study revealed a significantly stronger association of ADHD with suicidality in females than in males, although notably, the incidence rates of comorbid internalizing disorders (anxiety and depression) were higher in female participants (Ottosen et al. 2019). Regarding suicidal thoughts, one study reported no sex differences (Chronis‐Tuscano et al. 2010). Two studies reported data concerning suicide attempts and conflicting results: One study reported a greater risk in females (Chronis‐Tuscano et al. 2010), whereas the other reported a greater risk in males (Huang et al. 2018). In addition, one study reported a less pronounced risk in females than in males regarding thoughts or attempts at suicide (Lin et al. 2024).

### 5.2 ADHD Subtypes

Among the selected studies, two investigated different subtypes of ADHD (Chronis‐Tuscano et al. 2010; Swanson et al. 2012). One study reported an elevated risk for suicide ideation in individuals with the combined ADHD subtype compared with those with the inattentive and hyperactive subtypes (Chronis‐Tuscano et al. 2010). Similarly, the study reported an elevated risk for suicide attempt in the combined subtype but also in the hyperactive subtype (Chronis‐Tuscano et al. 2010). Another study reported an elevated rate of suicide attempts in the combined group compared with the inattentive group (Swanson et al. 2012).

### 5.3 The Role of Comorbidities

Eight studies reported data on follow‐up of the comorbid conditions, and three of these eight studies examined whether the comorbid condition had a mediating or direct effect on suicidal behavior (Chronis‐Tuscano et al. 2010; Swanson et al. 2012; Lin et al. 2024). One study identified depression as a statistically significant mediator of the relationship between ADHD and suicidal thoughts and attempts; however, the proportion of variance explained was relatively low (7.9%) (Lin et al. 2024). Another study reported a direct effect between ADHD and suicidality; however, there was a synergistic effect between generalized anxiety disorder, hypomanic episode, and substance‐related disorder (Chronis‐Tuscano et al. 2010). Finally, internalizing symptoms were a significant partial mediator in the remaining study (Swanson et al. 2012).

## 6 Discussion

To the best of our knowledge, this is the first meta‐analysis that has specifically examined the longitudinal relationship between ADHD diagnosed in children and adolescents and subsequent suicidal behavior. The current meta‐analysis included nine studies with a wide range of follow‐up periods: between 4 and 27 years (Chronis‐Tuscano et al. 2010; Hurtig et al. 2012; Huang et al. 2018; Swanson et al. 2012; Lin et al. 2024; Ottosen et al. 2019; Yoshimasu et al. 2019; Barbaresi et al. 2013; Sun et al. 2019). The majority of the studies used birth cohort data. We found an elevated risk for suicidality in all the variables (ideation, attempts, and death), with small‐to‐moderate effect sizes. Our results support the findings of previous systematic reviews from a longitudinal approach (James et al. 2004; Nigg 2013; Balazs and Kereszteny 2017; Impey and Heun 2012; Septier et al. 2019; Giupponi et al. 2018; Austgulen et al. 2023); that is, there is a significantly greater risk of suicide behavior in the ADHD population than in healthy controls, although it is important to note that the exact estimate should be interpreted with caution because the primary studies showed highly heterogeneous effect sizes (James et al. 2004; Nigg 2013; Balazs and Kereszteny 2017; Impey and Heun 2012; Septier et al. 2019; Giupponi et al. 2018; Austgulen et al. 2023). These results are also consistent with the previous meta‐analysis of Septier et al. (2019), which revealed a strong cross‐sectional relationship between ADHD and suicide behavior among youth and adults. In contrast to Septier et al. (2019), however, the present meta‐analysis also established temporal precedence in this relationship.

Additionally, an interesting preliminary result of the present meta‐analysis is that the risk might only present in the longer term; that is, only studies with a follow‐up time of 10 years or more showed a significant effect.

Compared to a previous systematic review published by Garas and Balazs (2020) on the topic, it is important to highlight several differences in the current meta‐analysis.

First, we updated the systematic review and included three additional studies that were published in the past 5 years. Additionally, we modified the inclusion criteria required for the meta‐analysis: specifically, we included only longitudinal studies where the clinical group was diagnosed with ADHD based on DSM‐5 criteria. During the selection process, we screened both the articles selected in our previous review and studies published in the past 5 years. Due to these differences in the inclusion criteria, only six studies out of the original 14 were included in the present study.

Furthermore, the narrative review of the nine studies highlighted that several factors might have an effect on the relationship between ADHD and suicidal behavior, including comorbid conditions, ADHD subtypes, and sex differences. This is in line with the literature. In a recent meta‐analysis, Liu et al. (2022) investigated the prevalence and correlates of suicidality in children and reported that ADHD and depression had the strongest correlations with suicidal ideation among different disorders. Furthermore, their data suggested that, among children, 5‐ to 14‐year‐old suicide decedents with known mental health problems more often experienced ADHD with or without hyperactivity than early adolescents did (Liu et al. 2022). There is also evidence that impulsivity in ADHD patients plays a role in the transition of suicidal thoughts to a death attempt (Ruch et al. 2021).

Recent studies have suggested that the most common comorbidities in ADHD patients are substance use disorders and mood disorders (Taurines et al. 2010). However, among youth hospitalized because of suicidal behavior, ADHD diagnoses were more common in children, whereas adolescents with mood disorders were more prevalent (Ben‐Yehuda et al. 2012). Another result suggests that it is possible that some of these adolescent patients have undiagnosed ADHD (Sandstrom et al. 2021). An interesting question is what role these comorbidities play in the occurrence of suicidal behavior in patients with ADHD (Balazs and Kereszteny 2017). Some of the studies included in our review suggested that disorders comorbid with ADHD, such as depression and other mood disorders, could play a partial mediating role in suicidality (Chronis‐Tuscano et al. 2010; Swanson et al. 2012; Lin et al. 2024). This finding is partially consistent with the previous finding of Levy et al. (2020), where total mediation of comorbid disorders was reported between ADHD and suicidality. Importantly, however, the study of Levy et al. (2020) was cross‐sectional and investigated children.

Regarding ADHD subtypes, the results of the two included papers were aligned in that suicide phenomena (both suicidal ideation and attempt) were more common in the ADHD combined subtype and the hyperactive/impulsive subtype than in the inattentive subtype (Chronis‐Tuscano et al. 2010; Swanson et al. 2012). A possible explanation may be the different characteristics of the ADHD‐C, ADHD‐HI, and ADHD‐I subtypes, for example, different symptoms (Association 2013), cognitive patterns (Li et al. 2024; Dovis et al. 2015), and neuroanatomy (Wu et al. 2022). In a recent study, the ADHD‐C group presented more behavioral problems, emotional lability, and less anxiety than the ADHD‐I group (Wu et al. 2022). In addition, impulsive and aggressive traits increase the risk of suicide in younger individuals (McGirr et al. 2008). Another finding is that ADHD‐C subtyped individuals are at the highest risk for both depression and suicide attempts (Chronis‐Tuscano et al. 2010).

In terms of sex differences, the sex proportions of the ADHD groups varied substantially in the primary studies (Chronis‐Tuscano et al. 2010; Huang et al. 2018; Lin et al. 2024; Ottosen et al. 2019). In the broader literature, the results are not homogenous in terms of sex differences in the ADHD population. In a recent follow‐up study with a mixed‐age ADHD sample (both adolescents and adults), a greater risk for suicidal behavior was found in females than males, but a greater risk of suicide death was reported in males than females (Fitzgerald et al. 2019), which is consistent with the findings of a previous birth cohort study (Ljung et al. 2014). Fewer data were available in the general population and in other pediatric populations (Liu et al. 2022), but the sex proportion might be consistent with that of the adolescent population; that is, suicidal thoughts and attempts are more frequent among women, whereas suicide death is more frequent among men (Cha et al. 2018).

A further interesting finding of our review is that no studies have focused on the severity indicators of ADHD (e.g., daily functionality, the number of symptoms, and their severity) when examining suicidal behavior. Importantly, however, the majority of the selected studies investigated data from medical records (Huang et al. 2018; Lin et al. 2024; Ottosen et al. 2019; Yoshimasu et al. 2019; Barbaresi et al. 2013; Sun et al. 2019). Symptom severity could be connected to externalized disorders (Barkley et al. 1990) and might cause severe peer relation problems (Bagwell et al. 2001). A study focused on ADHD trajectories reported that boys with moderate‐to‐high ADHD symptoms presented an increased risk of suicidal behavior in adolescence (Forte et al. 2020). This is an important direction for future research.

Notably, the results show great heterogeneity. On the one hand, this may be because of the great methodological heterogeneity of the studies; on the other hand, it seems likely that the ADHD population is not a homogenous population in terms of suicide risk. This may be because of the characteristics of the different ADHD subtypes, sex differences, and risk of suicide according to the severity of ADHD symptoms, in addition to the great heterogeneity of comorbidities and their combinations. In addition, there is also stress associated with life difficulties that appear or intensify with age.

During their development, children with ADHD face problems with social functioning, which are more pronounced in the case of persistent ADHD symptoms and behavioral disorders (Bagwell et al. 2001) and continue into adolescence. In addition to the normative difficulties arising from adolescence, the transition to adulthood seems to be more difficult in the case of ADHD, which is supported by a study reporting that suicide behavior is particularly pronounced at 20–29 years of age (Fitzgerald et al. 2019). Moreover, symptoms also change over time; adolescent girls with persistent ADHD have a greater risk for suicide attempts than transient ADHD patients do (Swanson et al. 2012). This is especially alarming because the prevalence of persistent ADHD in adulthood is 55% (Di Lorenzo et al. 2021).

Notably, we could not assess the role of SES. Furthermore, there might be other factors that should be considered. According to a recent meta‐analysis, child maltreatment and parental support are highly relevant to suicide ideation (Liu et al. 2022), which is related to insecure attachment (Wylock et al. 2023) and elevated parental stress, predominantly in the combined and hyperactive ADHD subtypes (Weinberger et al. 2018).

The low number of eligible studies also highlights the need for more longitudinal studies. Based on the current findings, we suggest the following methodological considerations for future studies: (1) Regarding the research design, cohort studies are advantageous for investigating temporal precedence; (2) in prospective cohort studies, several factors should be assessed, such as subtypes of ADHD, temporal stability, severity of symptoms, comorbid conditions, neglect, and parental support; (3) the time interval for assessing suicidal behavior is recommended to be longer (e.g., since baseline or lifetime); and (4) given that medication treatment could affect suicidal behavior (Man et al. 2017), this should also be taken into account.

The main implication of the current study for clinical practice is that there is a greater risk of suicide in ADHD patients. This is especially concerning considering that ADHD is still underdiagnosed in girls and older children. In addition to the early diagnosis of ADHD, the monitoring of suicidal behavior can be of particular importance in clinical work, especially in the case of parental neglect, comorbid mood disorders, and combined or hyperactive ADHD subtypes. Monitoring and appropriate treatment of comorbid symptoms might be particularly important in adolescence. During age transitions, the risk of suicidal behavior can increase; therefore, in addition to health care, it seems important to increase the social support of patients and improve the efficiency of self‐management and awareness of patients with ADHD.

## 7 Limitations

Our study has several limitations. First, due to the small number of available articles, the results are restricted by low statistical power, and we were unable to perform moderator analyses. As a result, precise conclusions regarding potentially key variables—such as ADHD subtypes, comorbidities, or medication use—could only be drawn through narrative analysis and is a serious limitation of the meta‐analytic results. Second, the studies differed in several methodological aspects, such as the duration of follow‐up, the confirmation of the ADHD persistence over time, and the measurement of suicidal behavior. All these could affect the results. Third, only a limited number of studies provided data regarding the factors that might affect the relationship between ADHD and suicidal behavior, such as ADHD severity and subtypes.

## 8 Conclusion

In summary, the current systematic review and meta‐analysis has confirmed previous findings that there is an elevated risk for suicidal behavior in ADHD patients and that this relationship is heterogeneous and complex, with important differences in ADHD subtypes across different ages, sexes, and comorbid mental and social problems, which are highly important in the development of suicidal behavior. Future research is needed to disentangle the complexity of this relationship.

## Author Contributions

Study concept and design: Garas, Takacs, Balazs Acquisition, analysis, or interpretation of data: Garas, Takacs, Balazs Drafting of the manuscript: Garas, Takacs Critical revision of the manuscript for important intellectual content: Garas, Takacs, Balazs Statistical analysis: Takacs Supervision: Balazs.

## Peer Review

The peer review history for this article is available at https://publons.com/publon/10.1002/brb3.70618

## Data Availability

The data that support the findings of this study are openly available on OSF at https://osf.io/w6hv3/.
